# Supplementary material for: Differential effect of heteronymous feedback from femoral nerve and quadriceps muscle stimulation onto soleus H-reflex
Source: PLoS One. 2023 Aug 14;18(8):e0290078. doi: 10.1371/journal.pone.0290078 (PMC10424854; doi:10.1371/journal.pone.0290078)
Supplement: S1 File — (DOCX) [file pone.0290078.s003.docx]

**Supporting Information**

Background EMG, stimulation evoked torque, and M- and H-wave sizes were not different across conditions.

The background SOL EMG prior to stimulation was consistently within the 6-10% MVIC target range for all the conditions. No differences in background SOL EMG were found between ISIs or stimulation locations (F_5,307_ = 0.46, *P*= 0.803). The background SOL EMG prior to stimulation was also not different for control and conditioned trials after collapsing across ISIs and stimulation locations (6.96 ± 1.32 %MVIC and 6.98 ± 1.34 %MVIC, for control and conditioned respectively, paired t-test: t_(14)_= -0.79059, *P* = 0.4448).

Stimulation intensities for Q (2x MT) and FN (1.6 ± 0.2 x motor threshold; range 1.5-2 x motor threshold) were chosen so that the peak twitch evoked knee extensor torques were matched across conditions within subjects. In support of this goal, torque was similar for stimulation location (i.e. FN and Q, F_1,146_ = 3.36, *P*= 0.07) and across ISI (F_5,146_ = 1.18, *P*= 0.32). Overall, torque values were 8.89 ± 1.87 Nm and 9.2 ± 2.05 Nm for FN and Q, respectively. Soleus M-wave size ranged between 1% and 13% of M_MAX_ across all ISI and stimulation methods. A valid SOL M-wave size could not be calculated when Q stimulation was applied during the ISI -8, -6, and -4 due to stimulation artifact in SOL EMG. Hence, only control M-wave sizes from the Q conditioning trials with the negative ISIs were used to evaluate M-wave consistency. The ANOVA examining SOL M-wave size showed no interaction or main effects of stimulation location (F_1,127_ = 0.29, *P* = 0.060) or ISI (F_5,127_ = 0.18, *P* = 0.972).

The H-reflex size during control trials was also monitored across stimulation locations and ISIs. H-reflex control size for FN and Q stimulation trials was 54.1± 7 and 56.4 ± 9.4 % H_MAX_, respectively. There were no significant differences in H-reflex control size across stimulation locations (2 levels: FN and Q, F_1,127_ = 3.88, *P* = 0.055) or ISI timings (6 levels: -8, -6, -4, 0, 20, 60, F_5,127_ = 0.54, P= 0.9749).
