# Supplementary material for: Differential effect of heteronymous feedback from femoral nerve and quadriceps muscle stimulation onto soleus H-reflex
Source: PLoS One. 2023 Aug 14;18(8):e0290078. doi: 10.1371/journal.pone.0290078 (PMC10424854; doi:10.1371/journal.pone.0290078)
Supplement: Attachment — Submitted filename: Response to Reviewers.docx [file pone.0290078.s004.docx]

**Reviewer #1: OVERALL IMPRESSION**
In this study, the authors compare in 15 healthy subjects the excitatory or inhibitory effects observed on the Soleus H-reflex, and the Soleus-EMG in a subgroup of 8 subjects, after electrical stimulation of either the Femoral Nerve or the Quadriceps.
The abstract is well constructed and correctly summarizes the study and its main findings.
In the introduction, the authors raise the question of the technical modalities to study specifically the heteronymous inhibitory circuits of the lower limbs.
The authors refer to previous studies focusing on muscle stimulation rather than on the femoral nerve in order to study more specifically the inhibitory circuits in patients who may present an exaggerated heteronymous excitation of soleus H-reflex.
Hypothesis are well made based on knowledge already acquired.
The figures are clear and accurate. The presentation makes the data easy to understand.
The results are clear and easily readable with the titles of the sub-paragraphs : « larger hetenomymous excitation was elicited with FN compared to Q stimulation (fig 2)», « FN and Q stimulation resulted in heteronymous inhibition of the SOL H-reflex (fig 3)» « heteronymous effects onto ongoing SOL EM of 10 and 20 % MVIC in subset of 8 participants ».
Then the correlations between the data received in H- reflex or EMG at 10 or 20% of the MVIC are given (heteronymous excitatory and inhibitory effects). The data are detailed.
The beginning of the discussion effectively summarizes the key data from this study: Consistent with the hypotheses and prior work [3, 15], heteronymous excitation elicited by FN stimulation was significantly larger than that elicited by Q stimulation (see Fig 2). Moreover, FN and Q stimulation 3elicited inhibition of the SOL H-reflex with similar magnitudes (see Fig 3). These findings suggest stimulation of the muscle belly can evoke similar heteronymous inhibition with less excitation compared to stimulation of the primary nerve trunk (i.e., FN).
The data are compared to previous studies.
Limitations of the study are discussed along with methodological considerations and functional implications.
Materials and methods : The procedures are clearly explained as well as the data analysis and the statistical analysis taking into account the post-hoc analysis.
Conclusions: considering these results the authors propose to use Q stimulation rather than FN stimulation to study heteronymous inhibition in the lower limb. One of the applications would be to use this method in patients with early heteronymous excitation e.g. post-stroke patients (ref 17).

**RESPONSE: We thank Reviewer 1 for his/her encouraging comments/impressions, thorough review, and constructive feedback. We feel that input improved the clarity of this revised manuscript.**

DISCUSSION OF SPECIFIC AREAS FOR IMPROVEMENT
No major issues

Minor issues : I just have minor issues to point out
1. Line 143-144 and fig 1 : when you look at the quadriceps stimulation electrodes on figure 1, you get the impression that they do not exactly match the text: « positioned on motor point areas distally (VM) and proximally –VL-RF). The authors should slightly modify the design of the electrodes position.
**RESPONSE: Thank you for bringing the misrepresented electrode positioning to our attention. We have significantly modified the figure to show the electrode positioning more clearly with a top-down view.**

2. Line 180 : what do the authors mean by « Q and FN conditioning trials were conducted on separate days » ? How long time between the two days ? Did they compare the Hmax/Mmax ratio between the 2 days ?
**RESPONSE: Thanks for the question and opportunity to clarify. In all but 1 participant, the 2 sessions were completed within a week of each other (range 1-9 days; mean 4.2 ± 2.8); One of the participants had sessions separated by 54 days due to unanticipated travel. We have added this information for clarification to the methods (Line 196-198)**

**We previously reported the H/M ratio average across days and have replaced with session 1 and session 2 values separately which were very close across days, see lines 198-199 (Day 1: 0.605 ± 0.13 and Day 2: 0.615 ± 0.14, paired t-test: p = 0.43)**

3. Line 200 : did the study of the influence of Q and FN stimulation onto EMG occur on the same days ? Could you clarify the time course?
**RESPONSE: The Q stimulation onto Soleus H-reflex and onto EMG occurred on the same day. The FN stimulation onto the Soleus H-reflex and onto EMG occurred on same day. However, the Q and FN stimulation conditioning sessions were completed on separate days as noted above.**

4. Line 413-415 : « The primary factors responsible for those exhibiting excitation due to Q stimulation remain to be identified. We speculate anatomical differences, such as more superficially positioned spindle axons, could be responsible. » Can you clarify: Is this just speculation or do you have any studies that can explain this?

**RESPONSE: Thank you for the question. We are unaware of any definitive physiological evidence or specific references. Bergquist et al (2011) have nicely reviewed the spatial distribution of axons in their figure 6. We have added this reference to this speculation sentence.**

**Reviewer #2: Comments to the Authors:**
The article entitled “Differential effect of heteronymous feedback from femoral nerve and quadriceps muscle stimulation onto Soleus H-reflex” is a research article investigating the effect of femoral nerve (FN) stimulation and quadriceps muscle belly (Q) stimulation onto soleus H-reflex and on-going EMG of soleus. The authors showed that FN stimulation elicited greater facilitation of H-reflex compared to Q stimulation. Similar effect was observed in on-going EMG of 20% maximal voluntary contraction (MVC), but not 10% MVC. Whereas heteronymous inhibition was elicited by FN stimulation and Q stimulation. From these results, the authors claim that Q stimulation is preferable choice to activate inhibitory pathway likely via Ib afferents.
The manuscript was unclear because many of measurement values did not describe in current version. So, it is difficult to support the authors’ claim. I suggest that the authors describe your data more precisely.
**RESPONSE: We thank reviewer 2 for his/her time and constructive comments. We feel your important comments and questions have resulted in a better and more clear manuscript. Our goal is to thoroughly describe our methods and results. We hope your concerns are clarified from the comments that follow.**

General Comments
1. To make it easier for non-experts to understand what was measured and analyzed, **I strongly suggest showing representative waveforms of H-reflex and on-going EMG.
RESPONSE: We have provided raw data waveforms as illustrative examples as suggested in Figure 1C and 1D.**

2. The authors described in statistical data only instead of raw data. I felt that many of individual raw data were ambiguous in this study (For example; knee extension torque induced by FN and Q stimulation, control H-reflex amplitude, M-wave amplitude of FN stimulation for MT…). These data will help replicate this study and will be beneficial in clarifying the results. Therefore, I suggest showing these measurement values.

**RESPONSE: We agree with the reviewer’s thoughtful comment. Our goal is to describe our methods and results to allow replication. We provide much of the data relevant to your question is in the supporting information document (i.e. supplementary material). Background activity during the H-reflex testing was consistent across conditions; Torque was not different between Q and FN stimulation conditions (9.2 +/- 2.05 Nm and 8.89 +/- 1.87 Nm, respectively;** **F_1,146_ = 3.36, *P*= 0.07). This information has been added to the supporting information document. Stimulation intensities as multiples of motor threshold were provided (Q=2xMT and FN=1.6) in the original submission. We have also provided stimulation currents in mA (Q stimulation= 46 ± 10.9 mA and FN stimulation: 20.7 ± 9.5 mA) in the revised version of the manuscript (see lines 187-188). We also provide information about the Soleus M-wave and the control H-reflex sizes (FN= 54% Hmax and Q=56% Hmax) in supporting information.**

3. The authors confirm the motor threshold (MT) while the knee flexed at 90° position (line 129), however, actual measurement was performed in 40° knee flexion and 60° hip flexion position. **It is speculated that this posture difference affects MT. Why did the authors select this position to measure the threshold?**
**RESPONSE: We appreciate your observation and acknowledge we wrote this in a confusing manner. We used the 90 degree position initially to assure the stimulation produced knee extension. We verified that the criterion (palpable contraction, visible M-wave, etc.) were met in the testing position. We have updated this paragraph in the methods.**

4. The authors described the MT of FN stimulation was the minimal current intensity that produced: 1) a visible M-wave on VL EMG, 2) palpable contraction in VL and VM, 3) accelerometer onset from VL below ≤20 ms from stimulation, and 4) visible motion of the hanging shank after stimulation (line 138) and the MT of Q stimulation was determined as the minimal current intensity that produced: 1) palpable contraction in VL, RF, and VM, with 2) visible motion of the shank after stimulation(line 147). How much the stimulus intensity did the authors actually used? In addition, what was the amplitude of the M-wave during FN and Q stimulation?
**RESPONSE: We thank the reviewer for the question and have provided the stimulation currents in mA in this revised version (see lines 187-188, Quadriceps stimulation: 46 ± 10.9 mA and femoral nerve stimulation: 20.7 ± 9.5 mA). Regarding the M-wave amplitude, unfortunately, we do not have this data. We did not record VL EMG during the quadriceps stimulation in part because of the large stimulus artifact from the doublet, making M-wave calculation difficult to achieve. We were also challenged to properly place VL surface EMG electrodes due to the large rectangular size relative to the morphology of some participants’ thighs. We agree having a comparison of M-wave size would be ideal; as an alternative, we controlled for the mechanical output of the muscle in response to the stimulation as knee extension torque (values provided in supporting information).**

5. Different stimulation pulses were used in this study, i.e., FN stimulation was used a single rectangular pulse with 1 ms duration, however, Q stimulation was used a paired pulse with 50 μs duration at 200 Hz. If the authors want to compare difference in stimulation sites, I suggest the authors should align the stimulus waveform. Did you perform that in preliminary experiment? It is unclear from the current results whether it is due to differences in stimulation sites or differences in stimulation waveforms.
**RESPONSE: We thank the reviewer for the question. The muscle stimulation parameters were chosen to reduce likelihood of activation of sensory axons as referenced in methods. The femoral nerve stimulation parameters were chosen based on the traditional approach used in many prior studies (e.g., Meunier et al. 1990; Dyer et al. 2014); thus, we feel this comparison is a valid test to address the study purpose. Future work should examine whether the effects observed due to muscle stimulation would differ using other stim parameters.**

**While we have not evaluated quadriceps muscle stimulation with 1 ms pulse width, sensory contributions from muscle stimulation evoked contractions has previously been reported to be much smaller compared to nerve stimulation in the quadriceps (Bergquist et al. 2012) and H-reflex responses are not readily elicited with muscle stimulation (e.g. Bergquist et al 2011; Bergquist et al. 2012; Nakagawa et al., 2020). Nonetheless, we agree future work should examine the relative influence of pulse width in the methodological considerations section; In addition, we include and acknowledge evidence in which stimulation with 1 ms pulse widths of other muscle nerves (medial gatsroc, soleus) can elicit heteronymous responses attributed to sensory axons (e.g,. Horslen et al. 2017; Pierrot-Deseilligny et al. 1981). Please see end of methodological considerations paragraph in discussion (Lines 521-532)**

6. It was difficult to understand the contents of the figures. What does the boxplot mean? What does the dot? What did the authors compare? Please add a more detailed explanation.
**RESPONSE: We thank the reviewer for identifying this oversight. We have included a description in each of the figure captions describing what the boxplot and dots represent (i.e., box represents quartiles of data, horizontal line reflects median, and each dot is individual participant data).**

7. The authors compared the results of H-reflex and on-going EMG, however the background EMG level was quite different (H-reflex, 6~10% of MVC [line 156]; on-going EMG, 10 and 20% of MVC [lines 205, 207]). I suggest examining and comparing the effects on the H-reflex when the amount of contraction is changed.
**RESPONSE: We appreciate the comment. The H-reflex size is a common dependent variable used to examine the strength of a reflex pathway between two muscles. The H-reflex size recorded during a low level of background EMG (6-10%), as used in the present study, is advocated and a common approach used by many researchers (Knikou 2008; Theodosiadou et al. 2023; Stein and Thompson, 2006). Ongoing EMG is also a common dependent variable used when examining the strength of a reflex pathway between muscles. The influence of heteronymous feedback from femoral nerve onto ongoing soleus emg has been evaluated at 20% MVC (Lyle et al, 2022) and 30% MVC (Dyer et al 2014; Meunier et al. 1996). The motivation to use 10% MVC in this study was to identify whether a lower physical effort (10 vs 20% MVC) could be used to quantify excitatory and inhibitory feedback. A lower physical effort of 10% would be preferable for patient populations with residual muscle weakness. These patients can have a hard time sustaining a 20% or greater MVC.**

**We feel our comparison of the heteronymous effects from femoral nerve/quadriceps muscle stimulation onto the H-reflex size and ongoing EMG as described in this study are consistent with best practices. While H-reflex size could be evaluated at higher background levels in future studies, we feel that our methodological approach should adhere to best practice principles. Apologies if we do not fully understand your comment.**

8. The authors claimed that the heteronymous inhibition induced by Q stimulation was similar to that by FN stimulation (Figure 4). However, Q stimulation has a large outlier at each 10% and 20% MVC (Figure 4B). Because the number of participants was small (8 participants, line 201), I feel that the results of one person have a large impact on the author's claim. If exclude this data, it may appear that there is difference in inhibition area elicited by FN stimulation and Q stimulation similar to H-reflex experiment. Therefore, it is difficult to support the authors’ claim from the current result.

**RESPONSE: We appreciate the comment and agree heterogeneity with a small sample can influence results. In relation to H-reflex comparisons, the inhibition was similar between 20 and 60 ms ISI (though inhibition from FN stimulation was larger than Q at 0 ms ISI). Therefore, we have made this explicit in first paragraph of discussion.**

**In relation to heteronymous effects onto EMG, we have added a sentence in the results expanding upon significant main effect of stimulation location. Femoral nerve inhibition was greater than quadriceps per significant main effect. The pairwise comparisons were not different statistically possibly due to sample size and because 1 participant had larger inhibition relative to the sample for both FN and Q. We acknowledge in the discussion (methodological considerations paragraph) that the small sample size is a limitation. We do state in the discussion that overall FN caused larger inhibition than Q (see line 461-467); the statistical analysis revealed the difference was due to the 0 ms ISI with no differences found between 20 and 60 ms ISI.**
