# Supplementary material for: Differential effect of heteronymous feedback from femoral nerve and quadriceps muscle stimulation onto soleus H-reflex
Source: PLoS One. 2023 Aug 14;18(8):e0290078. doi: 10.1371/journal.pone.0290078 (PMC10424854; doi:10.1371/journal.pone.0290078)
Supplement: Attachment — Submitted filename: Response to Reviewers_Revision2.docx [file pone.0290078.s005.docx]

Review Comments to the Author

Reviewer #1: I thank the authors for answering my questions and requests for clarification.
The manuscript is now quite clear and deserves publication in an international journal.

**RESPONSE: We thank reviewer 1 for their constructive review.**

Reviewer #2: Comments to the Authors:
The authors have made many of the changes I suggested and the quality of the manuscript is improved, but several minor issues remain:

**RESPONSE: We appreciate the positive feedback and your constructive review.**

**General Comments**
The authors mentioned that excitation and inhibition elicited by FN and Q stimulation was detected by using pre-stimulus background EMG and ±1SD values (line 245~). In addition, the onset latency of excitation/inhibition was set based on the previous reports. However, I think that there are many cases where excitation/inhibition are induced other than the specified latency such as Figure 1C (Quadriceps conditioning; inhibitory like response at -30 ms, facilitatory like response at 15~20 ms).

**RESPONSE: We thank the reviewer for the question. In our methods, we indicate the latency criterion (excitation onset >23 ms and inhibition onset <45 ms post the electrical stimulation) we used that is based on conduction times previously established by other authors. An additional criterion was that the excitatory and inhibitory responses had to last at least 2 ms. The “inhibitory like response at -30 ms” did not last 2 ms. The “quadriceps conditioning facilitatory like response at 15-20 ms” the reviewer questioned in Fig 2C is a stimulus artifact harmonic that occasionally happens. The three peaks you note during this region do not stay above the 1SD line for 2 ms and occur prior to the earliest possible neural effects (3^rd^ peak at 17 ms) from activating muscle spindle axons. We have added new labels on the figure and text in the caption describing the stimulation artifact.**

What did the authors do if the reflex was not induced at the desired latency? Did the authors measure several times? Or increase the number of stimulation and averaged? Was 1SD the appropriate for this study (Lines 247, 252)?
**RESPONSE: Facilitatory and inhibitory reflex responses were quantified as described in the methods (i.e., excitatory: soleus EMG exceeding 1 SD above the mean background for at least 2 ms so long as after 23 ms from stimulation). We are not sure exactly what is meant by “measured several times” or “increase the number of stimulation and averaged”? The analyses were completed on the rectified soleus EMG as an ensemble average of 20 repetitions. We feel the 1 SD and 2 ms period was appropriate.**

**For reference from methods (lines 254-261): “Excitation onset was determined as the time point when the SOL EMG trace exceeded 1SD above the mean for ≥ 2 ms. The end of excitation was determined as the time at which the SOL EMG trace returned below the 1 SD line for a period of ≥ 2 ms. Only excitatory responses with onset ≥ 23 ms were considered as arising from heteronymous Ia facilitation [1, 5]. Inhibition onset and termination were determined as the SOL EMG moving 1SD below the mean background SOL EMG for a period of ≥ 2 ms and returning above the 1 SD line for ≥ 2 ms. Inhibitory responses were considered in analysis only if the onset was < 45 ms since the fastest transcortical effects could manifest soon thereafter [15].”**

Specific Comments
Line 315, 335, 357: What is “a participant”? Should explain more precisely.

**RESPONSE: The comment appears to refer to our figure captions where we use the phrase “each dot represents a participant.” We use the term participant to indicate a person that was recruited and participated in this study. We have modified the phrase to read “each dot represents an individual participant” to hopefully make clear that the data point dots represent separate persons that participated in the study.**

Line 482: Does “GTO” mean “Golgi tendon organs”?

**RESPONSE: Thank you for identifying the use of the abbreviation without defining this clearly in the text. We have written Golgi tendon organ for all occurrences instead of the abbreviation on lines 62 and 503.**

Line 422: I cannot follow the author’s claim that Ia afferents contribute the facilitation in the current version. Please describe more why the authors considered Ia afferents contribute to the facilitation in terms that non-experts can understand.

**RESPONSE: The comment is in a paragraph of the discussion that is discussing potential reasons for some participants exhibiting excitation due to quadriceps stimulation. The most widely accepted origin of excitation is from muscle spindle feedback (see Line 437: “Heteronymous excitation elicited by FN stimulation arises from activating muscle spindle axons with monosynaptic projections onto SOL motoneurons [1, 5].” We have added some additional text in the paragraph referenced in your comment and in several other paragraphs (e.g., lines 208-210; 415-417) to help explain for non-experts.**

Line 462: How did the previous reports reveal the contribution of Ib afferents to the inhibition? Please describe more why the authors considered the recurrent inhibition and Ib inhibition contribute to the inhibition in terms that non-experts can understand.

**RESPONSE: The papers cited are a mix of animal and human studies; we feel the methodological details are difficult to summarize for non-experts due to several approaches (e.g., nerve stimulation and recording from motoneurons in decerebrate cat; controlled pairwise muscle stretches of 2 muscles in the decerebrate cat) and beyond the scope of this paper. We feel the main ideas that apply here are that Golgi tendon organ Ib sensory axons or motor axons (evoke recurrent inhibition) can be activated directly by the stimulation current and the twitch contraction can mechanically activate Golgi tendon organ receptors. We have added text we hope helps non-experts better understand the distinction between effects elicited by current acting on sensory/motor axons and mechanical activation of Golgi tendon organ receptors (e.g. Lines 480-489).**

Figure 1A: The configuration of stimulation electrodes is difficult to understand from this picture. Does this picture only show the cathode? Where is the anode?

**RESPONSE: Sorry for the confusion. We specifically added a top-down view to make the electrode configuration more clear. The cathode electrode is shown for the femoral nerve electrode but the anode is positioned on the posterior buttock and not shown. We have added a note in the caption about not including the anode here. Two electrodes show the positioning of quadriceps muscle belly stimulation electrodes on the figure and are described in the caption as dotted lines. The cathode and anode specification for the quadriceps was based on the lowest intensity that resulted in a contraction of both VM and VL/RF. We have added this text to the methods section.**

Figure 1B: The authors showed the representative waveforms elicited by FN stimulation. Please show additional waveforms elicited by Q stimulation.

**RESPONSE: We have added Q stimulation waveforms to Figure 1B.**

Figure 1B: I think there is a stimulus artifact due to the conditioning stimulation (especially, left waveform; ISI -6 ms). Is it correct?

**RESPONSE: The figure shows a stimulus artifact from the tibial nerve stimulation. There is not a clear stimulus artifact seen on this waveform from the FN stimulation which is typical. Please see the Quadriceps stimulation excitatory waveform has a visible stimulation artifact since the stimuli occur just after the tibial nerve stimulation.**

Figure 2A: I think the horizontal axis is not correct. The authors should describe that the ISI is different for the FN stimulation and Q stimulation as in lines 207~212.

**RESPONSE: We have added text to the caption to make clear that Q stimuli were applied 2.5 ms before and after the FN single stimulus for each ISI.**

Figure 4: Please add the representative waveforms for 10% and 20% MVIC conditions.

**RESPONSE: Since representative waveforms illustrating the FN and Q stimulation conditions at 20% MVIC are shown in Fig 1C, we prefer to not include more waveforms here.**
